# Artificial Intelligence in the Assessment and Management of Spasticity: A Scoping Review

**DOI:** 10.3390/bioengineering13101209

**Published:** 2026-10-16

**Authors:** Ruoxi Cao, Ruojin Li, Joon Sin Ser, Yeow Leng Tan, Jing Chen

**Affiliations:** 1 Department of Rehabilitation Medicine, Singapore General Hospital, Singapore 169608, Singapore; liruojin@gt.cn (R.L.); joonsin.ser@mohh.com.sg (J.S.S.); tan.yeow.leng@singhealth.com.sg (Y.L.T.); 2 Department of Rehabilitation Medicine, Beijing Electric Power Hospital of State Grid Company of China, Beijing 100073, China; 3 Rehabilitation Medicine, SingHealth Residency, Singapore 168582, Singapore

**Keywords:** spasticity, artificial intelligence, machine learning, rehabilitation, scoping review

## Abstract

Spasticity is a prevalent and disabling consequence of upper motor neuron disorders, yet its assessment and management remains clinically challenging. Artificial intelligence (AI) may offer opportunities to enhance the objective assessment, prediction, and management of spasticity; however, the current evidence base has not been comprehensively characterised. This review systematically mapped evidence on the application of AI across the continuum of spasticity care and identified key evidence gaps to inform future research. PubMed, Embase, CINAHL, and Web of Science were searched for peer-reviewed English-language studies evaluating AI in adult spasticity. Two reviewers independently screened studies, extracted data, and assessed methodological quality using NHLBI tools and PROBAST + AI. This scoping review was prospectively registered with the Open Science Framework Registries. The search yielded 1832 records, of which 21 studies were included after screening. One additional study was identified through citation searching, bringing the total to 22 studies involving 1114 participants. Applications spanned four clinical domains: discrimination between spastic and healthy individuals (*n* = 2), severity assessment (*n* = 18), risk prediction (*n* = 1), and treatment response prediction (*n* = 1). Most studies focused on post-stroke upper limb spasticity. Surface electromyography was the most frequently used input modality, often integrated with kinematic or kinetic sensors. Conventional machine learning predominated, with reported accuracies of 74–96% and the Modified Ashworth Scale as the most common reference standard. All 22 studies were at high overall risk of bias on PROBAST + AI, and none performed external validation, highlighting substantial methodological limitations that constrain clinical translation.

## 1. Introduction

Spasticity is a prevalent motor disorder following upper motor neuron lesions, representing a primary driver of long-term disability in neurorehabilitation [1]. It affects approximately 30% to 42.6% of patients within 3 to 6 months after stroke [2,3], and is similarly observed in approximately 40–78% of individuals with spinal cord injury (SCI) [4,5], 70–80% of those with cerebral palsy (CP) [6], 60–84% of patients with multiple sclerosis (MS) [7], and 20–50% of traumatic brain injury (TBI) survivors [8].

The conceptualisation of spasticity has undergone significant refinement over the last four decades. The classic definition by Lance highlighted velocity-dependent augmentation of tonic stretch reflexes and stretch reflex hyperexcitability [9]. Subsequently, Pandyan et al. proposed a broader conceptual framework and defined spasticity as “disordered sensorimotor control, resulting from an upper motor neuron lesion, presenting as intermittent or sustained involuntary activation of muscles” [10]. The 2017 European consensus further clarified that velocity-dependent stretch reflex hyperresponsiveness is a core feature of spasticity [11]. Most recently, the American Academy of Physical Medicine and Rehabilitation consensus in 2024 expanded this conceptualisation by integrating both neural and non-neural contributors, defining spasticity as a disorder of sensorimotor control characterised by velocity- and muscle length-dependent involuntary muscle overactivity and increased resistance to externally imposed muscle stretch [12]. Building on this perspective, current expert frameworks increasingly recognise the clinical heterogeneity of spasticity, including distinct manifestations such as spasms [13]. This paradigm shift underscores the need for more refined assessment approaches and targeted intervention to support individualised, function-oriented management. Accordingly, there is a clear clinical need for more quantitative assessment tools that enable the objective measurement of spasticity and provide a reliable basis to guide subsequent management.

The assessment of spasticity remains clinically challenging because of its complex and dynamic nature. The Modified Ashworth Scale (MAS), although the most widely used tool in clinical practice, has well-documented limitations, including poor inter-rater reliability—particularly for mid-range scores—and weak correlation with functional outcomes [14,15,16]. Furthermore, the MAS does not differentiate neural components of spasticity from non-neural contributors such as soft tissue contracture, nor does it permit continuous or dynamic biomechanical monitoring [17]. Alternative measures, such as the Tardieu Scale, attempt to better characterise velocity-dependent responses and partially distinguish neural from non-neural components; however, they remain constrained by ordinal scoring systems and inherent subjectivity in clinical interpretation [18]. Collectively, these tools fail to reflect the continuous, context-dependent, and dynamic nature of spasticity [1].

Beyond the measurement challenges, the lack of robust evidence-based management strategies continues to complicate clinical decision-making in spasticity care. Currently, there are limited tools available to predict spasticity progression, and treatment approaches remain insufficiently standardised, with no universally accepted guidelines, thereby compromising clinical outcomes. Addressing these interconnected challenges necessitates a shift toward dynamic, data-driven approaches in neurorehabilitation [10,12,17,19].

In this context, artificial intelligence (AI)-driven approaches have emerged as a promising strategy, leveraging their capacity to integrate multimodal clinical, biomechanical, and neurophysiological data, capture nonlinear and time-varying patterns of motor impairment, and facilitate the continuous monitoring of spasticity-related motor dynamics [20,21]. These strengths of AI align with core demands of spasticity management, particularly in addressing clinical heterogeneity, improving characterisation, and supporting personalised treatment strategies—key needs that conventional tools cannot sufficiently meet. Recent investigations have focused on fusing multimodal sensor-acquired data—especially surface electromyography (sEMG) and inertial measurement units (IMUs)—with AI algorithms, with the MAS commonly employed as the clinical reference standard for model validation [22,23,24]. These integrated AI approaches also enable the objective and quantitative characterisation of spasticity. Compared to conventional MAS-based assessments, AI-driven strategies provide superior reproducibility, improved quantitative precision, and lower subjective rater bias [23,25].

However, no systematic or scoping review has yet comprehensively synthesised and mapped AI applications within a full-cycle spasticity care continuum, thereby offering a roadmap for integrating AI into clinical workflows. In response to these identified gaps, this review aims to systematically map the existing evidence on the application of AI across the continuum of spasticity care—encompassing prediction, assessment, and management. Specifically, three primary objectives guide this review: (1) to characterise the predominant AI methodologies and data modalities currently employed in spasticity research; (2) to critically appraise the developmental stages and level of clinical translation of these AI-enabled approaches; and (3) to identify key evidence gaps that can inform future research priorities and accelerate the clinical implementation of AI-driven spasticity assessment and management strategies.

## 2. Materials and Methods

This scoping review was conducted and reported in accordance with the Preferred Reporting Items for Systematic Reviews and Meta-Analyses extension for Scoping Reviews (PRISMA-ScR) [26]. The review protocol was prospectively registered with the Open Science Framework Registries (https://osf.io/cqd7m, accessed on 1 March 2026).

### 2.1. Eligibility Criteria

We included all studies reporting the use of AI or machine learning for spasticity assessment, prediction, or management in adults (≥18 years) with upper motor neuron lesions or neurologic conditions, including but not limited to stroke, CP, SCI, TBI, and MS. The inclusion criteria were as follows: (1) interventional and observational studies (e.g., randomised controlled trials [RCTs], cohort, case–control, and cross-sectional studies), as well as pilot/feasibility and algorithm validation studies; (2) spasticity explicitly assessed, measured, or analysed as an outcome; (3) AI or machine learning used as a core analytical method; (4) human studies with at least five participants; and (5) peer-reviewed full articles published in English.

The exclusion criteria were as follows: (1) participants aged under 18 years or with unclear age without separate adult data; (2) focus on non-spastic hypertonia or spasticity mentioned without formal assessment; (3) no AI or machine learning methods, conventional statistics only, or rule-based algorithms without autonomous learning; (4) AI applications without spasticity-specific outcomes; (5) use of synthetic or simulated data only, or animal and cadaver studies; (6) case reports, conference abstracts, reviews, editorials, letters, and protocols without empirical results.

### 2.2. Search Strategy

The search strategy was designed to identify peer-reviewed studies published in English. A preliminary pilot search was conducted in PubMed to identify key articles relevant to the topic. Terms and index terms (e.g., MeSH terms) were extracted from the titles and abstracts of these articles and used to develop a comprehensive search strategy, which was subsequently adapted for Embase, CINAHL, and Web of Science. The full search strategy was tailored for each database. Searches were conducted from database inception to 6 March 2026.

A structured query was developed to identify studies containing at least one term related to spasticity and one term related to AI in the title or abstract. The detailed search strategy is presented in Appendix A Table A1.

### 2.3. Selection Process

After duplicate removal, two reviewers independently screened titles and abstracts according to predefined eligibility criteria (Appendix A Table A2). Full texts of potentially relevant studies were retrieved and assessed independently by both reviewers.

Discrepancies at any stage were resolved through discussion, with arbitration by a third reviewer when necessary. The study selection process was documented using a PRISMA flow diagram.

To verify the comprehensiveness of the electronic search, we conducted a manual check of the reference lists of the included studies and relevant reviews, as well as a supplementary forward citation search. Two reviewers independently screened the results.

### 2.4. Data Extraction

A standardised data extraction form was developed, informed by the PCC (Population, Concept, Context) framework and study objectives. Two reviewers independently conducted a pilot extraction to assess the form’s clarity and completeness. The form was subsequently refined prior to formal data extraction.

During the formal phase, duplicate data extraction was performed independently by two reviewers. Extracted variables included study characteristics (e.g., study design, sample size, and population), AI-related components (e.g., input data, algorithms, and validation methods), and model performance and clinical agreement (e.g., accuracy, area under the curve [AUC], and reference standards).

All extracted data were cross-checked by both reviewers prior to finalisation. Discrepancies were resolved through discussion, with a third reviewer consulted where consensus could not be reached.

### 2.5. Evaluation of Study Quality

Study quality was appraised across two domains: clinical methodological quality and AI model risk of bias.

Clinical methodological quality was evaluated independently by two reviewers using the National Heart, Lung, and Blood Institute (NHLBI) Quality Assessment Tools, selected for their applicability across diverse study designs [27]. The NHLBI tool for Controlled Intervention Studies was applied to the single RCT, while the remaining observational studies were assessed using the NHLBI tool for Observational Cohort and Cross-Sectional Studies. Each study was rated across 14 criteria, with responses recorded as “Yes,” “No,” “Cannot Determine,” “Not Applicable,” or “Not Reported,” and summarised as overall quality ratings of “Good,” “Fair,” or “Poor.”

AI-specific risk of bias was assessed by using the Prediction model Risk Of Bias Assessment Tool for Artificial Intelligence (PROBAST + AI) [28]. This framework evaluates risk of bias across four domains: participants, predictors, outcomes, and analysis. Each domain was rated as “low,” “high,” or “unclear” risk of bias, from which an overall risk of bias judgement was derived.

### 2.6. Data Synthesis

A narrative synthesis was conducted because of heterogeneity in study designs, AI methodologies, and outcome measures, which precluded meta-analysis. Included studies were summarised descriptively and presented in tabular format (Tables 1–3), focusing on study characteristics, AI methodology, model performance, interpretability, and limitations.

## 3. Results

### 3.1. Study Selection

**Figure 1:**
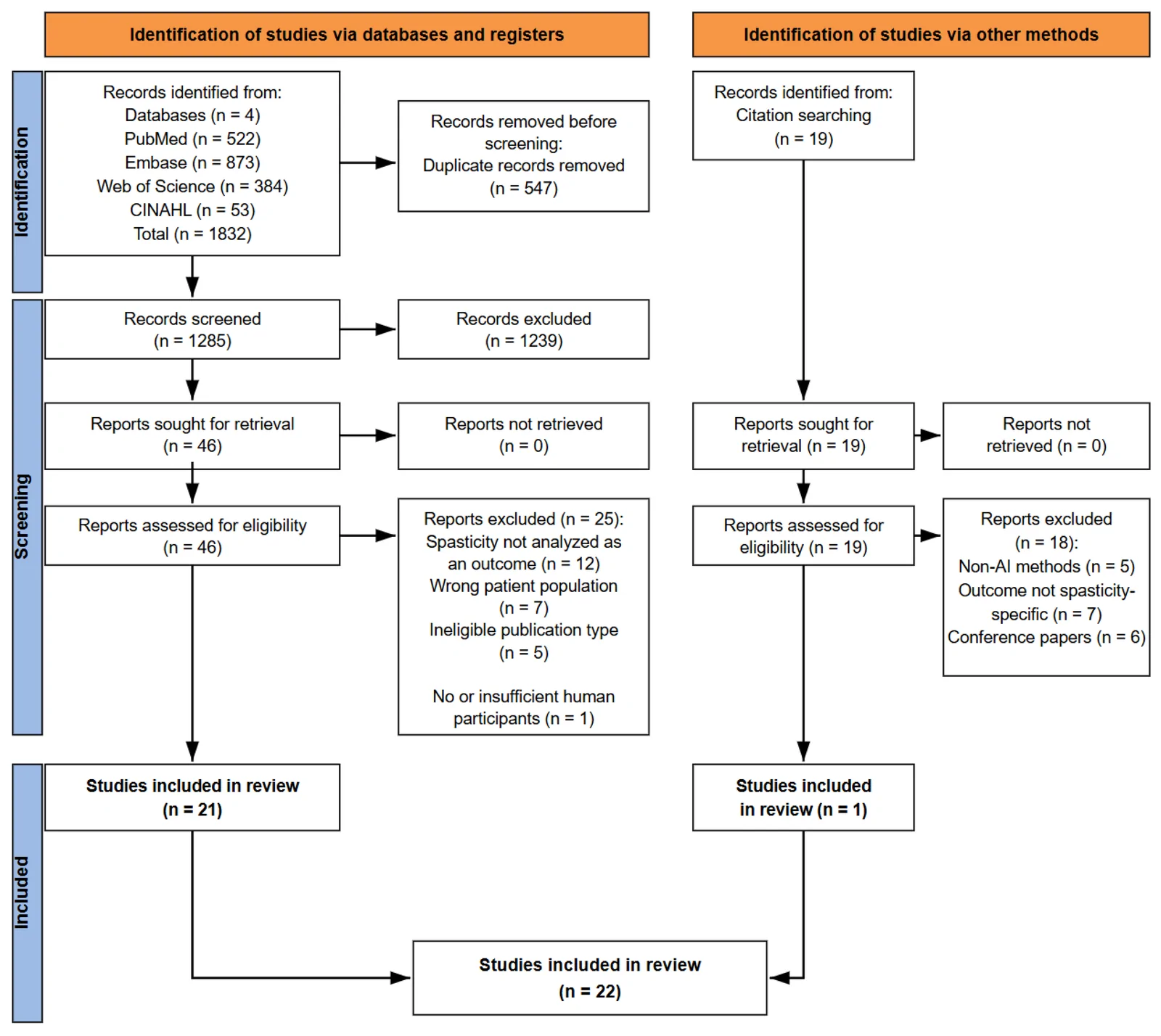
PRISMA flow diagram.

A total of 1832 records were identified from the initial search using the strategy outlined in Appendix A Table A1. After removing duplicates, 1285 records remained for title and abstract screening. Of these, 1239 records were excluded, and 46 full-text articles were assessed for eligibility based on the PCC framework (Appendix A Table A2). Following full-text review, 21 studies met the predefined eligibility criteria and were included in this scoping review. In addition, a manual check of the reference lists of the included studies and forward citation searching were performed to identify additional eligible studies. This process identified 19 potentially relevant records, of which 18 were excluded (non-AI methods, *n* = 5; outcome not spasticity-specific, *n* = 7; conference papers, *n* = 6). One additional study met the eligibility criteria and was included, bringing the total number of included studies to 22. The study selection process is illustrated in the PRISMA flow diagram (Figure 1).

### 3.2. Study Characteristics

**Figure 2:**
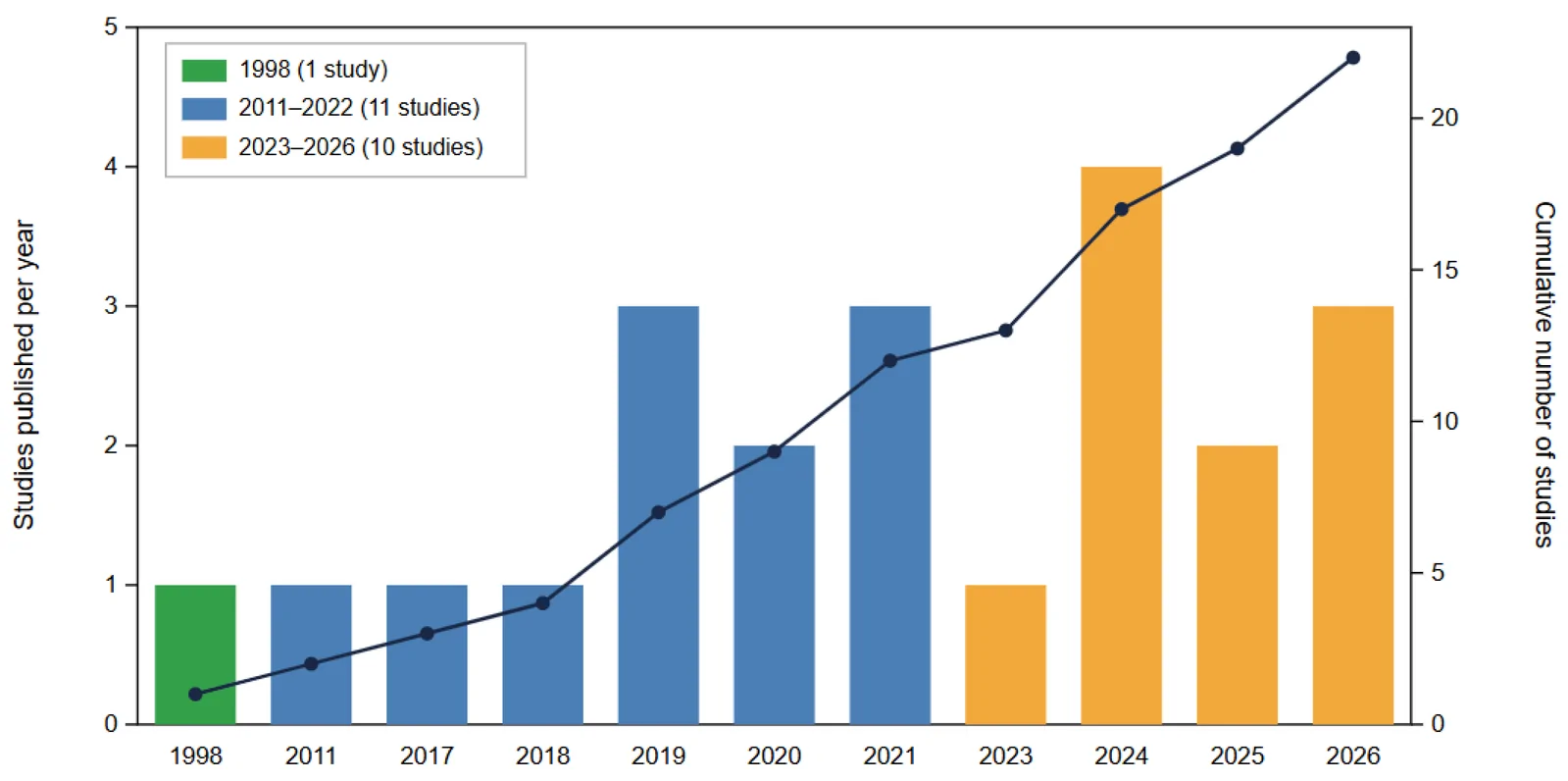
Publication trends of included studies (*n* = 22). Bars show the number of studies published per year, coloured by period (2011–2022 in blue, 2023–2026 in orange). The line shows the cumulative number of studies over time. Publication output increased markedly from 2023 onward, with 10 of 22 studies (45%) published between 2023 and 2026.

A summary of study characteristics, including designs, sample sizes, populations, etiologies, body parts assessed, and MAS grades, is presented in Table 1. One study was published in 1998; the remaining 21 were published between 2011 and 2026, of which 10 were published between 2023 and 2026 (Figure 2). Twelve studies were conducted in China (including Taiwan and Hong Kong), with the remainder originating from Malaysia, South Korea, Canada, Brazil, Slovenia and Turkey. Study designs included 21 observational studies (20 cross-sectional [22,24,25,29,30,31,32,33,34,35,36,37,38,39,40,41,42,43,44,45] and one prospective cohort [46]) and one randomised controlled trial [47].

A total of 1114 participants were included across the 22 studies. Of these, 21 studies (1089 participants) provided detailed participant counts for each aetiology. Among these individuals, 787 had stroke, 24 had TBI, 106 had SCI, 2 had CP, 3 presented with other rare neurological disorders, and 167 were healthy controls. One study involving 25 participants reported only broad diagnostic categories (stroke, TBI, CP) without exact numbers [32]. Sample sizes ranged from 11 to 200 participants. Overall, sample sizes were generally small: 16 studies enrolled fewer than 60 participants, whereas only two studies included more than 100 cases. MAS grades across the included studies ranged from 0 to 4. All 22 studies included grade 2, 21 included grade 1, 17 included grade 3, 12 included grade 0, and 7 included grade 4.

Data collection focused predominantly on upper limb spasticity (*n* = 18). Only three studies investigated lower limb spasticity [31,43,45], and one study assessed both upper and lower limbs [47].

Most studies used the MAS as the clinical reference standard or classification label. Of the 18 severity assessment studies, 15 used MAS as the sole reference standard, while two also incorporated the Fugl–Meyer Assessment (FMA) as a measure of motor function.

### 3.3. Clinical Applications of AI in Spasticity

A detailed summary of AI methodologies, input data modalities, outputs, and validation methods for each clinical application is presented in Table 2 and Table 3. The included studies were categorised into four application domains: discrimination between spastic and healthy individuals (*n* = 2), assessment of spasticity severity (*n* = 18), prediction of future spasticity risk (*n* = 1), and prediction of response to spasticity treatment (*n* = 1).

#### 3.3.1. Discrimination Between Spastic and Healthy Individuals

Two cross-sectional studies addressed discrimination between spastic and healthy individuals (Seth 2015, *n* = 85; Alcan 2017, *n* = 24) [30,31]. Study populations included individuals with stroke or TBI alongside healthy controls. Both studies used unimodal features but differed in signal sources: one used sEMG (neurogenic features), while the other employed a robotic system to collect biomechanical features. Both studies demonstrated strong discriminatory performance, with reported accuracies ranging from 85% to 96%, sensitivity reaching 100%, and specificity up to 94%.

A key insight from the kinematic-based model was its ability to detect subtle motor control abnormalities even in individuals classified as MAS 0, suggesting potential for identifying subclinical spasticity not captured by conventional clinical scales.

Both studies had small sample sizes and no external validation, and one used a complex robotic setup that may limit clinical accessibility.

#### 3.3.2. Assessment of Spasticity Severity

Eighteen cross-sectional studies addressed severity assessment, representing the most common clinical application [22,24,25,29,32,33,34,35,36,37,38,39,40,41,42,43,44,45]. Study populations predominantly comprised individuals with post-stroke spasticity, with some studies (*n* = 6) including SCI, TBI, and CP [25,32,33,34,35,45]. Sample sizes ranged from 11 to 98 participants, with 14 enrolling fewer than 50 patients. Only one old study used AS as the reference standard, all the remaining 17 studies used MAS as the reference standard; two also incorporated the FMA for motor function [22,41], and four incorporated the TSRT [29,36,38,40]. Data collection focused predominantly on upper limb spasticity (*n* = 16), with only two study investigating lower limb involvement [43,45].

Of the 18 included studies, data modalities were predominantly unimodal (*n* = 13), while five adopted multimodal approaches integrating both biomechanical and neurogenic-relevant modalities [25,34,37,38,40]. Among neurophysiological modalities, sEMG was used in 11 studies (6 unimodal and 5 multimodal), while EIM was used in one unimodal study to assess muscle bioelectrical properties [44]. Biomechanical modalities were reported in 11 studies (6 unimodal and 5 multimodal), with kinematic and kinetic parameters derived from IMUs, pressure sensors, mechanomyography (MMG), and a triboelectric soft pneumatic actuator (TENG-SPA) that captured resistance during spastic finger stretching [41].

Fourteen studies focused on classification of spasticity severity, reporting accuracies of 74–95%, with the highest F1-score of 0.95, indicating strong agreement with clinical ratings. The lower bound of this range reflects Zupan et al. (1998), the earliest study in this review, which used C4.5 decision trees and k-nearest neighbours to predict Ashworth Scale (AS) scores from sEMG features and reported accuracies of 74–76% [45]. In multi-joint classification tasks (elbow, wrist, thumb, and fingers) [24], F1-scores ranged from 0.90 to 0.95, suggesting consistent performance across anatomical regions.

Four studies developed continuous spasticity indices, demonstrating strong correlations with MAS (R^2^ up to 0.96). These findings suggest that continuous measures may provide greater sensitivity than ordinal MAS grades. Among studies reporting correlation metrics, AI-derived indices showed strong positive correlations with MAS scores (r or R^2^ = 0.85–0.96) and strong negative correlations with upper extremity function measured by FMA (r > 0.90), indicating good alignment with clinical and functional outcomes.

However, of these studies, 13 excluded patients with MAS 4 and 5 excluded those with MAS 3—4. Twelve studies included patients with MAS 0 in their cohort, while six did not. Only three studies included the full MAS range of 0 to 4 [25,35,41]; one further study used the original AS across the full 0–4 range [45]. No study performed external validation.

#### 3.3.3. Prediction of Future Risk of Spasticity

Only one of the 22 included studies addressed prediction of future spasticity risk, a prospective cohort by Chen L et al. [46]. The study enrolled 172 first-ever stroke patients and used routine clinical data collected within 10 days post-stroke, including the National Institutes of Health Stroke Scale (NIHSS), FMA, modified Rankin Scale (mRS), and modified Barthel Index (mBI), to predict development of spasticity at six months. Machine learning models achieved AUC values up to 0.98, with the FMA Upper Extremity score identified as the strongest predictor. This approach has high clinical feasibility as it uses readily available clinical variables without specialised equipment, potentially enabling early identification of patients at risk for targeted preventive interventions. Limitations include single-centre design, lack of external validation, and exclusion of patients with severe cognitive deficits.

#### 3.3.4. Prediction of Response to Spasticity Treatment

Only one of the 22 included studies addressed prediction of treatment response, an RCT by Chen H et al. [47]. The study enrolled 200 post-stroke patients receiving botulinum toxin type A combined with vibration therapy, using clinical scales, demographics, and ultrasound biomarkers (muscle thickness, echo intensity) as predictors. Of these, 192 completed the 12-week follow-up and were included in the final analysis. Neural network and ensemble models achieved an AUC of 0.87 for predicting treatment response at 12 weeks, with muscle ultrasound features identified as important predictors guiding treatment decisions. Unlike risk prediction studies using routine clinical data, this trial incorporated ultrasound biomarkers to predict treatment response, representing a novel multimodal approach for personalised rehabilitation. This approach could support personalised spasticity management by identifying patients likely to benefit from specific interventions. Limitations include single-centre design, lack of external validation, modest sample size for deep learning approaches, and a 12-week follow-up that may not capture long-term treatment effects.

### 3.4. AI Methodology and Performance

**Figure 3:**
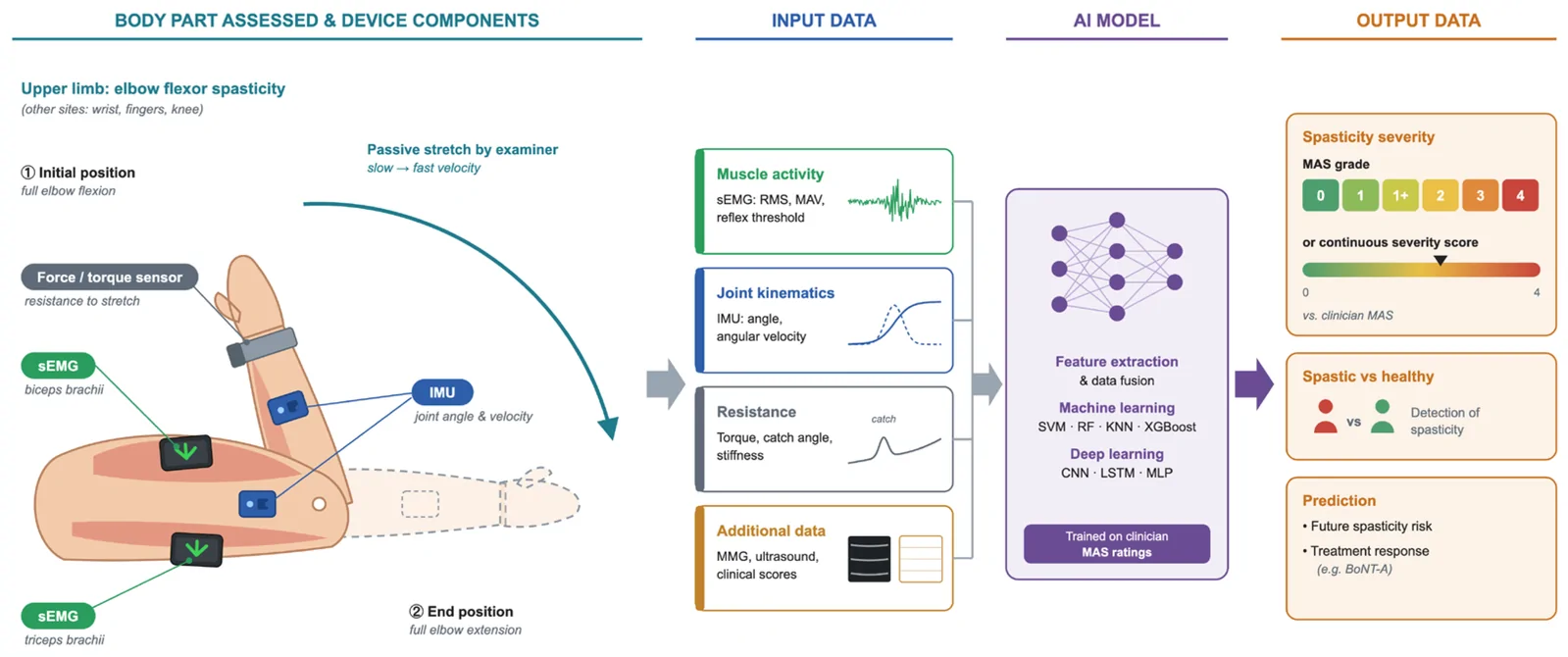
AI methodology pipeline synthesised from the 22 included studies. The schematic illustrates a representative AI-based spasticity assessment workflow: (1) body part assessed and device components; (2) input data, including sEMG, kinematics, ultrasound, and clinical measures; (3) AI model, comprising feature extraction and data fusion, conventional machine learning, and deep learning approaches trained on clinician MAS ratings; and (4) output data, including spasticity severity, discrimination between spastic and healthy individuals, and prediction of future spasticity risk or treatment response. Abbreviations: AI, artificial intelligence; BoNT-A, botulinum toxin type A; CNN, convolutional neural network; IMU, inertial measurement unit; KNN, k-nearest neighbours; LSTM, long short-term memory; MAV, mean absolute value; MAS, Modified Ashworth Scale; MLP, multilayer perceptron; MMG, mechanomyography; RF, random forest; RMS, root mean square; sEMG, surface electromyography; SVM, support vector machine; XGBoost, Extreme Gradient Boosting.

To provide an overview of the methodological landscape across the 22 included studies, Figure 3 presents a schematic overview of a representative AI-based spasticity assessment pipeline synthesised from the included studies, illustrating the typical body part assessed, device components, input data modalities, AI models, and output types. Validation approaches varied across studies. External validation was absent (0/22). Internal validation methods included k-fold cross-validation (*n* = 12), leave-one-out or leave-one-subject-out cross-validation (*n* = 6), train/test split only (*n* = 1), and internal validation without cross-validation (*n* = 3).

**Figure 4:**
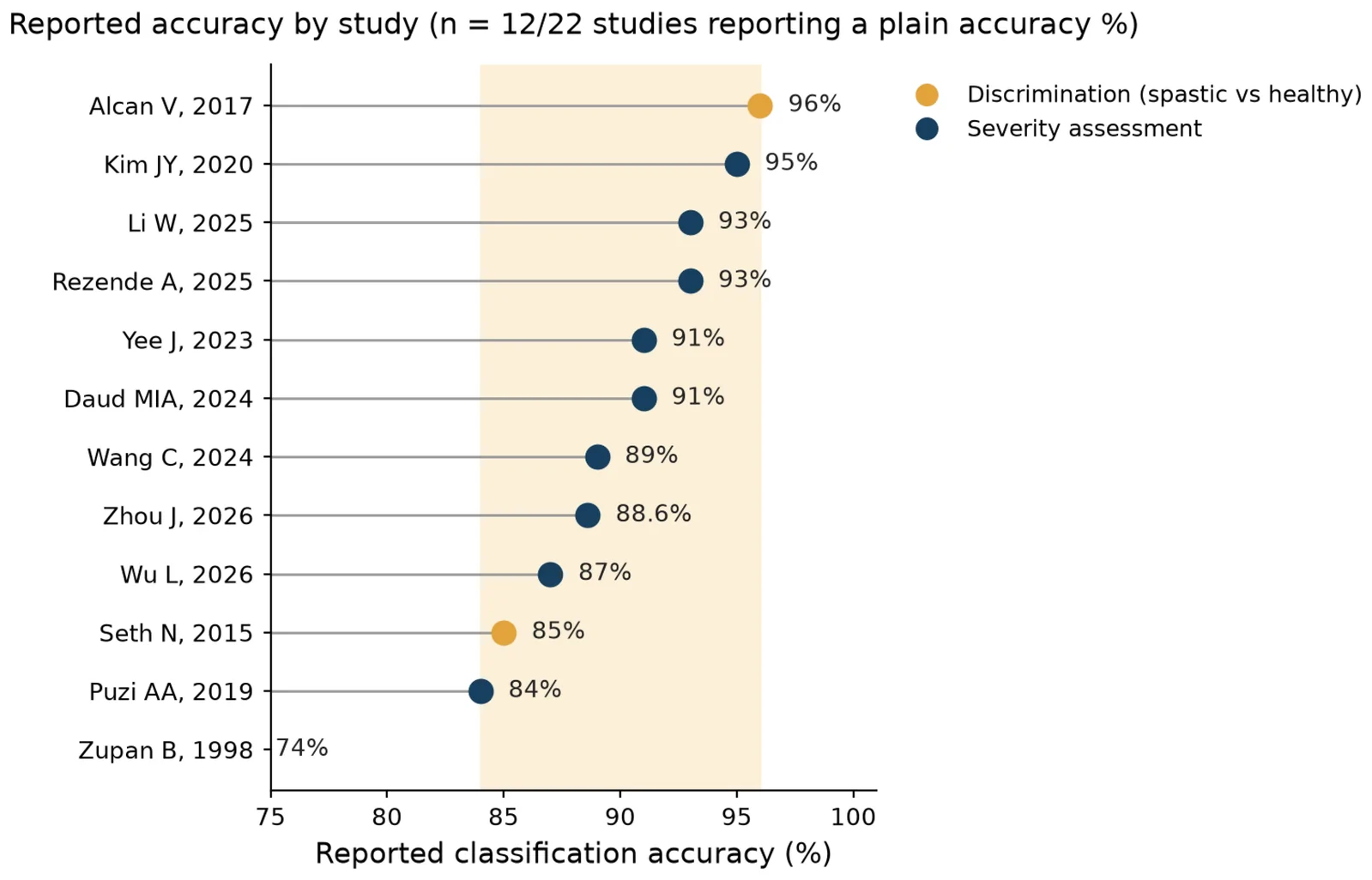
Reported classification accuracy by study (*n* = 12 of 22 studies reporting a plain accuracy percentage) [25,30,31,32,35,39,40,41,42,43,44,45]. Orange dots represent discrimination studies (spastic vs. healthy); blue dots represent severity assessment studies. The shaded band represents the 84–96% range within which most included studies reported classification accuracy. Zupan et al. [45] reported a lower accuracy (75.6%), consistent with its early machine-learning methods.

Among the 22 included studies, conventional machine learning (CML) was the most frequently used approach (*n* = 18, 81.8%). CML-only models were reported in 15 studies. Across the 12 studies that reported a plain accuracy percentage, classification accuracies ranged from 74% to 96% (Figure 4). Commonly used algorithms include support vector machines (SVMs), random forests (RFs), k-nearest neighbours (KNN), and Extreme Gradient Boosting (XGBoost). Three CML + DL studies, respectively, reported: (1) 88.9% accuracy with Long Short-Term Memory-Support Vector Regression (LSTM-SVR) (R = 0.86 with MAS); (2) AUC = 0.87 for treatment response prediction (neural network); and (3) 88.6% accuracy for distinguishing stroke patients from healthy controls using Impedance Myography Circle Mapping (IMCM) parameters (R^2^ > 0.87 with MAS) [40,44,47]. Two DL-only model studies were identified: one reported an accuracy of 93%, and the other reported 82% agreement with human assessors [33,41]. Employed DL architectures included convolutional neural networks (CNNs), long short-term memory networks (LSTMs), multilayer perceptrons (MLPs), and feedforward neural networks (FFNNs). Fuzzy logic classifiers (FLCs) were used in two studies, both employing adaptive neuro-fuzzy inference systems (ANFISs), achieving strong regression performance with R^2^ values ranging from 0.82 to 0.96 [31,36].

Of these studies, multimodal approaches were adopted in six studies. Five combined neurogenic and biomechanical features, all within the severity assessment domain, where classification accuracy ranged from 89% to 91%, F1-scores reached up to 0.95, and regression R^2^ values ranged from 0.86 to 0.93 [25,34,37,38,40]. The remaining multimodal study integrated clinical indicators with ultrasound biomarkers and addressed treatment response prediction, reporting an AUC of 0.87 [47]. Among 16 unimodal studies, neurogenic features alone (*n* = 8, including sEMG and EIM) achieved 74–96% accuracy and R^2^ = 0.80–0.96; biomechanical features alone (*n* = 7, including kinematic, kinetic, MMG, and TENG-SPA) achieved 82–95% accuracy, 0.90–0.95 F1-score, and R^2^ = 0.85–0.88 (Kappa = 0.74 in one study); clinical indicators alone (*n* = 1) addressed risk prediction and achieved AUC = 0.98 [46].

Given the comparable performance between multimodal and unimodal approaches, sEMG was the most frequent input modality, appearing in 12 studies. Among these, seven studies used sEMG alone as a unimodal neurogenic feature source, extracting time-domain metrics (root mean square, mean absolute value) and frequency-domain features (wavelet-based measures) [22,29,31,36,42,43,45]. The remaining five studies adopted multimodal approaches that combined sEMG with IMUs (for joint angles and movement velocity), force/torque sensors, or pressure balls to capture biomechanical features, thereby enabling a comprehensive assessment of spasticity. In terms of performance, the highest reported accuracy (96%) was achieved by a unimodal sEMG study performing binary discrimination (healthy vs. post-stroke) [31].

For the challenging task of spasticity grading (ordinal multi-class classification), multimodal sEMG-based approaches reported accuracies of 89–91% in the two studies that performed classification [25,40], while the remaining studies reported F1-scores up to 0.95 [37] and regression R^2^ values of 0.86–0.93 [34,38]. Unimodal sEMG studies reported accuracies of 87–93% in severity grading tasks [42,43], with one study reporting R^2^ up to 0.96 [36] and another reporting correlation coefficients of 0.80–0.92 [22]. Direct comparison between multimodal and unimodal approaches was limited by heterogeneity in reported performance metrics. Models were classified according to a standardised taxonomy in healthcare AI into three categories: intrinsic interpretable models, post hoc explained models, and black-box models [48]. Classification was based on the primary interpretability approach described in each study. Eight studies employed intrinsically interpretable models, including fuzzy logic systems, linear algorithms, and rule-based methods, achieving accuracy between 74% and 96% and r/R^2^ values ranging from 0.93 to 0.96 [29,30,31,32,34,36,39]. Four studies applied post hoc explainability techniques—such as SHAP, layer-wise relevance propagation, and feature importance analysis—and reported accuracy between 82% and 91%, with AUC/Kappa values of 0.74–0.86 [24,25,33,45]. Notably, ten studies (including both deep learning and conventional machine learning approaches) relied exclusively on black-box models without any interpretability analysis; this group attained accuracy of 87–95%, AUC/F1 scores of 0.87–0.95, and r/R^2^ values of 0.80–0.92 [22,35,37,38,40,41,42,43,44,46].

### 3.5. Risk of Bias Assessment

The methodological quality of included studies was assessed using the NHLBI tools (Table 4), while AI-specific risk of bias was evaluated using the PROBAST + AI framework (Appendix A Table A3).

Based on NHLBI criteria, one RCT was rated as “Good” [47], while the remaining 21 observational studies were rated as “Fair.” Across observational studies, all clearly defined research questions, study populations, and outcome measures (Q1, Q2, Q4, Q8, Q11 = Y). However, none reported sample size justifications (Q5 = N), 20 did not provide participation rates (Q3 = NR) and report blinding of outcome assessors (Q12 = NR), and fewer performed statistical adjustment for confounding variables (Q14 = Y for only 4 studies, including the RCT) [24,34,46,47].

**Figure 5:**
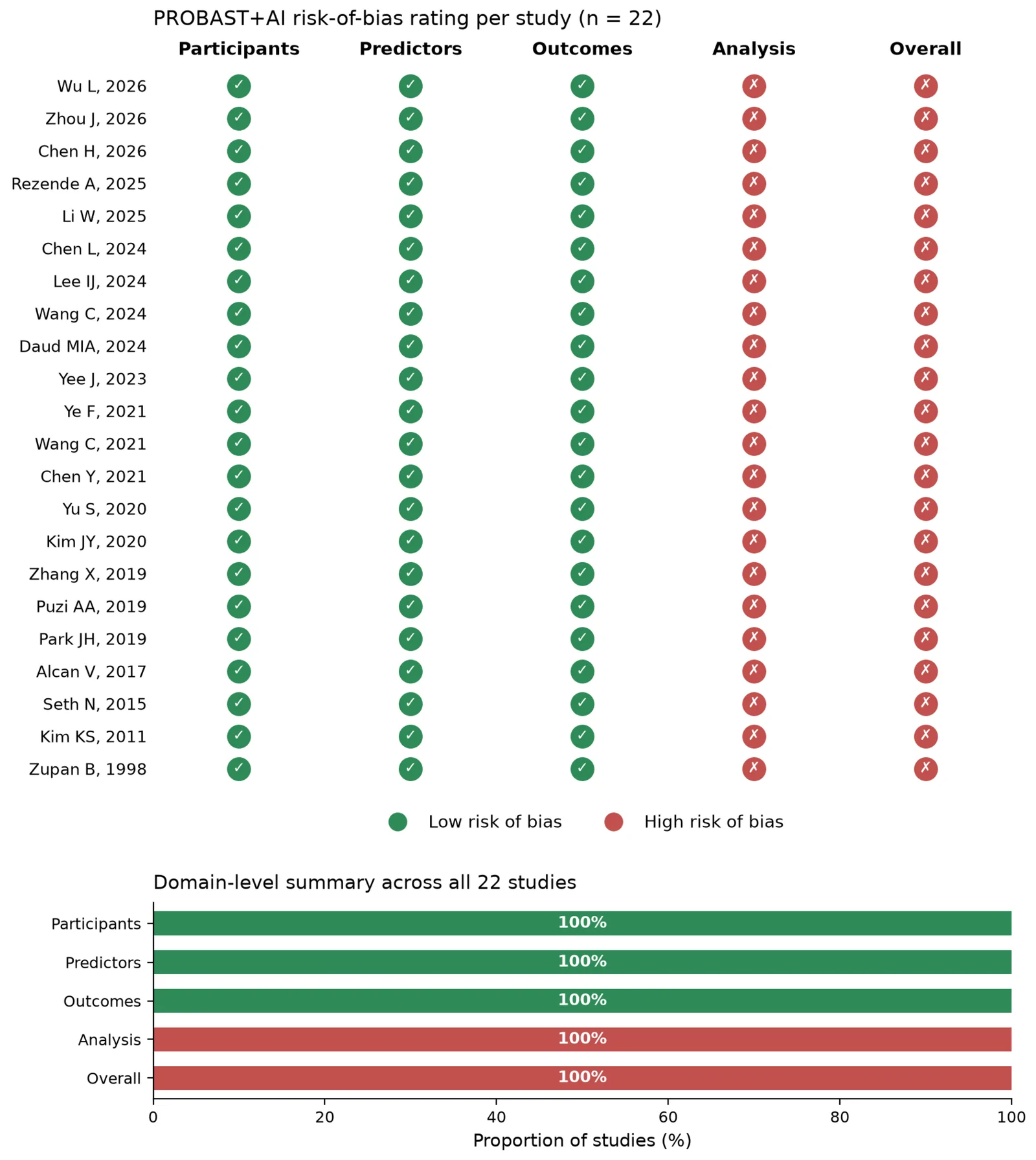
PROBAST + AI risk-of-bias assessment across the 22 included studies [22,24,25,29,30,31,32,33,34,35,36,37,38,39,40,41,42,43,44,45,46,47]. (**Top panel**): domain-level ratings for each study (green = low risk of bias; red = high risk of bias). All studies were rated low risk in the Participants, Predictors, and Outcomes domains, and high risk in the Analysis domain, resulting in an overall high risk of bias for all studies. (**Bottom panel**): domain-level summary, showing the proportion of studies rated low or high risk within each domain.

Assessment using PROBAST + AI showed that all 22 studies were at overall high risk of bias. All studies demonstrated low risk in the Participants, Predictors, and Outcomes domains, and high risk in the Analysis domain (Figure 5). Table A3 in the Appendix A provides the detailed explanatory notes for each study’s Analysis-domain rating. Common issues in the Analysis domain included small sample sizes relative to model complexity, lack of external validation, potential information leakage (e.g., preprocessing prior to data splitting), absence of model calibration assessment, class imbalance without mitigation strategies, and use of overlapping time-series windows or repeated measures without accounting for within-subject dependence. These findings indicate that methodological limitations in model development and validation were widespread across the included studies, substantially constraining the current evidence base for clinical translation.

## 4. Discussion

To our knowledge, this scoping review is the first to systematically map the application of AI across the full continuum of spasticity care. The current evidence can be broadly categorised into four domains: (1) discrimination between spastic and healthy individuals; (2) assessment of spasticity severity; (3) prediction of future spasticity risk; and (4) prediction of response to spasticity treatment. Among these, the majority of studies (18 of 22) focus on severity assessment, reflecting a strong emphasis on quantitative evaluation. Surface electromyography, often combined with kinematic and kinetic data, was the most commonly used input modality. Overall, the findings indicate that AI-based methods can achieve high levels of diagnostic precision and demonstrate promising predictive performance in spasticity assessment and management. Collectively, these results underscore the potential of AI to shift current practice from subjective, ordinal clinical grading toward objective, data-driven, and precision-oriented frameworks.

### 4.1. Multimodal AI Approaches and Clinical Utility

A key finding of this review is that many AI systems are not merely automating MAS scoring, but may instead capture distinct neurophysiological and biomechanical dimensions of spasticity. Contemporary conceptual frameworks increasingly recognise spasticity as a multidimensional disorder involving both neural and non-neural contributors, including reflex hyperexcitability, altered muscle activation, viscoelastic stiffness, contracture, and impaired motor control [9,11,12,13]. Conventional bedside assessment tools incompletely distinguish these components and have additional limitations, including poor inter-rater reliability, which may contribute to suboptimal treatment decisions and inadequate longitudinal monitoring [17,49]. In contrast, multimodal AI approaches integrating neurogenic signals (e.g., sEMG) with biomechanical measurements (e.g., velocity, torque, resistance, joint motion) may better reflect the composite nature of spasticity [25,37,38,40]. This distinction is clinically important because resistance to passive movement may arise from different underlying mechanisms requiring different therapeutic strategies. AI-driven continuous modelling therefore has potential not only to improve quantification, but also to support more refined, mechanism-targeted rehabilitation interventions. In practice, such systems could potentially support more standardised longitudinal monitoring, guide treatment selection, improve injection targeting, or identify patients requiring escalation of spasticity management.

Neurogenic features derived from sEMG provide information regarding reflex-mediated muscle activation and abnormal motor unit recruitment, whereas kinematic and kinetic sensors quantify movement resistance, velocity dependence, and passive tissue properties. Their integration may therefore better approximate the multidimensional construct recognised in contemporary definitions of spasticity. In this review, 6 of 22 studies adopted multimodal approaches [25,34,37,38,40,47], with several demonstrating high performance [34,37]. However, unimodal approaches also achieved comparable performance in terms of both classification accuracy [41,42] and correlation with the MAS [22,24,36], suggesting that the added value of multimodal integration may extend beyond improving conventional performance metrics. Potential advantages such as robustness, cross-subject generalisation, and treatment response prediction were rarely evaluated as primary outcomes in the included studies. Thus, the extent to which multimodal integration improves performance or clinical utility beyond unimodal approaches remains uncertain. The immediate priority may therefore be less about increasing model complexity and more about establishing how these approaches can be integrated into clinical workflows, inform treatment decisions, and ultimately improve patient outcomes.

The uneven distribution of studies across clinical application domains is itself a key finding of this review. Severity assessment dominated (*n* = 18), whereas discrimination between spastic and healthy individuals (*n* = 2), prediction of future spasticity risk (*n* = 1), and prediction of treatment response (*n* = 1) were each represented by only one to two studies. This distribution reflects the current state of the field, in which AI research has concentrated on severity assessment, and highlights areas where evidence remains scarce. The predominance of assessment-focused studies is consistent with the clinical priority of objective spasticity assessment, given the subjectivity and limited inter-rater reliability of conventional scales such as the MAS. In contrast, risk and treatment response prediction require longitudinal, multicentre, and multimodal data, and remain at an early stage of evidence accumulation.

### 4.2. Interpretability and Clinical Trust

Another key insight from this review is that high model performance does not require black-box architectures. Intrinsically interpretable models (fuzzy logic systems, decision trees, linear regression) appeared capable of achieving performance within a similar range to black-box models (accuracy 74–96% vs. 87–95%), although direct comparisons were limited by study heterogeneity. This finding aligns with Ennab and Mcheick [47], who highlighted concerns regarding the limited interpretability of some AI models in healthcare, as outputs that cannot be readily understood or scrutinised by healthcare providers may increase the risk of misinterpretation and inappropriate treatment decisions. Rosenbacke et al. [50] further demonstrated that explainable AI enhances clinician trust, while complex or contradictory explanations may undermine it. In rehabilitation settings, where clinical decisions are often longitudinal, multidisciplinary, and highly individualised, transparent and interpretable decision support systems may be particularly important. Accordingly, the marginal accuracy gains of black-box models may not justify their reduced transparency.

### 4.3. Methodological Limitations and Risk of Bias

Despite the promising performance reported across studies, an important conceptual limitation must be acknowledged. Most AI systems were trained and validated using the MAS as the primary reference standard despite the recognised psychometric limitations of the MAS itself. High agreement with MAS scores therefore does not necessarily indicate accurate physiological quantification of spasticity. Rather, AI models may partially reproduce the inherent subjectivity and construct limitations embedded within the reference scale. This may create a form of circular validation, in which model performance is evaluated primarily against an imperfect clinical reference standard. Future studies should therefore move beyond exclusive reliance on MAS-based supervision and incorporate multidimensional comparators, including biomechanical metrics, neurophysiological measures, functional outcomes, patient-reported outcomes, and real-world activity monitoring.

The findings of this review also highlight a persistent “validation gap” between current AI research and clinical readiness. Across all 22 included studies, no external validation was performed. External validation is essential because it tests whether a model developed on one dataset generalises to different populations, settings, and measurement protocols [50]. Without it, reported performance metrics may substantially overestimate true model accuracy when deployed in new clinical environments [51]. The lack of external validation is particularly concerning given the heterogeneity of rehabilitation populations and assessment conditions. Sensor placement, movement velocity, therapist handling, acquisition protocols, and patient-specific motor patterns may all influence signal characteristics. Consequently, models developed under highly controlled laboratory conditions may not perform similarly in routine clinical environments. Bridging this gap will require prospective studies that validate AI models in routine clinical settings with representative patient populations.

Additionally, small sample sizes (15 of 22 studies enrolled fewer than 50 participants) increase overfitting risk, particularly for deep learning architectures [52]. Overfitting occurs when a model learns noise and idiosyncrasies specific to the training dataset rather than generalisable features of spasticity, leading to artificially inflated performance estimates that are unlikely to replicate in new patients [52]. Application of the PROBAST + AI framework suggested that all included studies were at overall high risk of bias, driven primarily by issues within the analysis domain [28]. This pattern of suboptimal quality appraisal is not unique to our review. Morone et al. similarly found that less than half of systematic reviews on AI in clinical medicine used dedicated tools for assessing AI-specific metrics [53]. Addressing these interconnected limitations will be essential to translate promising technologies from proof-of-concept studies into clinically viable tools.

### 4.4. Priorities for Future Research

Several priorities emerge for future research (Table 5). First, external validation on independent, multicentre datasets is urgently needed to assess generalisability across diverse populations and clinical settings [51]. Future studies should also include broader patient cohorts, particularly individuals with lower limb spasticity and non-stroke etiologies such as SCI, MS, and CP, as current evidence is heavily weighted toward post-stroke upper limb spasticity. Collaborative consortia and data sharing initiatives could facilitate the collection of larger and more diverse datasets. Second, larger prospective studies with adequate statistical power are required. Sample size calculations should account for model complexity to avoid overfitting [52]. Future research should prioritise longitudinal datasets and outcome prediction over cross-sectional proof-of-concept studies. Third, real-time implementation and integration into clinical workflows should be prioritised, including user-friendly interfaces and electronic health record integration [54]. Looking forward, the integration of machine learning with real-time simulation has been proposed as a key direction for enhancing personalised spasticity assessment and treatment [49]. Fourth, the development of interpretable AI models is critical for clinical trust. Given that intrinsically interpretable models achieved comparable accuracy to black-box models in the included studies, transparency may be achievable without sacrificing performance. When black-box models are necessary, post hoc explainability analyses should be routinely applied and reported [55,56]. Such transparency may support clinical validation and trust, and could become increasingly relevant as regulatory frameworks for AI in healthcare evolve [54,57]. Finally, research should be extended to under-represented clinical domains beyond severity assessment.

### 4.5. Strengths and Limitations

This review has several strengths. The search strategy was comprehensive, covering multiple electronic databases with an independently peer-reviewed strategy. Detailed data extraction enabled comprehensive mapping of the evidence base. Risk of bias was assessed using both NHLBI tools and the PROBAST + AI framework [28].

Limitations include restriction to English-language articles, possible publication bias [58], heterogeneity precluding meta-analysis, and exclusion of grey literature and preprints, which may contain relevant emerging research. Additionally, the geographic concentration of included studies may limit the generalisability of our findings. This distribution reflects the geographic distribution of AI research in spasticity rather than a selection bias, as no geographic filters were applied. Future studies should include more diverse populations to ensure that AI models are validated across different ethnic, cultural, and healthcare settings.

## 5. Conclusions

In conclusion, this scoping review highlights that AI is facilitating a shift from subjective, ordinal clinical grading toward more objective, data-driven approaches in spasticity assessment and management. AI-based methods have demonstrated high accuracy in distinguishing spastic from non-spastic muscles, quantifying severity using continuous measures, and predicting both future risk of spasticity development and treatment response. However, high performance alone does not equate to clinical readiness. All 22 included studies were at high overall risk of bias and none performed external validation, reflecting a substantial ‘validation gap’ between reported performance and clinical applicability. To enable meaningful clinical implementation, future research should prioritise explainable AI approaches, larger and more diverse study populations, and robust external validation within multicentre settings. If these methodological and translational challenges can be addressed, AI-based approaches may become valuable adjuncts for more objective, individualised, and data-driven spasticity management.

## Figures and Tables

**Table 1:** Characteristics of the 22 included studies.

First Author, Year	Location	Study Design	Sample Size (*n*)	Aetiology of Spasticity *	Body Part Assessed	MAS Grades of Patient Cohort
Wu L, 2026 [43]	China	Cross-sectional	11	Stroke (*n* = 11)	Lower limb	1–3
Zhou J, 2026 [44]	China	Cross-sectional	83	Stroke (*n* = 50), Healthy controls (*n* = 33)	Upper limb	1–4
Chen H, 2026 [47]	China	RCT	200	Stroke (*n* = 200)	Upper and lower limbs	1+ to 4
Rezende A, 2025 [42]	Brazil	Cross-sectional	15	Stroke (*n* = 10), Healthy controls (*n* = 5)	Upper limb	1–3
Li W, 2025 [41]	China	Cross-sectional	25	Stroke (*n* = 15), Healthy controls (*n* = 10)	Upper limb	0–4
Chen L, 2024 [46]	China	Prospective cohort	172	Stroke (*n* = 172)	Upper limb	0–4
Lee IJ, 2024 [24]	Taiwan	Cross-sectional	62	Stroke (*n* = 40), Healthy controls (*n* = 22)	Upper limb	0–2
Wang C, 2024 [40]	China	Cross-sectional	30	Stroke (*n* = 20), Healthy controls (*n* = 10)	Upper limb	1–3
Daud MIA, 2024 [39]	Malaysia	Cross-sectional	30	Stroke (*n* = 30)	Upper limb	0–3
Yee J, 2023 [25]	Malaysia	Cross-sectional	50	Stroke (*n* = 41), TBI (*n* = 3), CP (*n* = 2), Cerebral meningitis (*n* = 1), HIE (*n* = 1), SCI (*n* = 2)	Upper limb	0–4
Ye F, 2021 [22]	Hong Kong	Cross-sectional	29	Stroke (*n* = 29)	Upper limb	0–3
Wang C, 2021 [38]	China	Cross-sectional	26	Stroke (*n* = 16), Healthy controls (*n* = 10)	Upper limb	0–3
Chen Y, 2021 [37]	China	Cross-sectional	13	Stroke (*n* = 9), Healthy controls (*n* = 4)	Upper limb	1–2
Yu S, 2020 [36]	China	Cross-sectional	15	Stroke (*n* = 11), Healthy controls (n = 4)	Upper limb	1–2
Kim JY, 2020 [35]	South Korea	Cross-sectional	48	Stroke (*n* = 44), SCI (*n* = 4)	Upper limb	0–4
Zhang X, 2019 [34]	China	Cross-sectional	24	Stroke (*n* = 13), SCI (*n* = 2), TBI (*n* = 1),Healthy controls (*n* = 8)	Upper limb	1–3
Puzi AA, 2019 [32]	Malaysia	Cross-sectional	25	Stroke, TBI, CP (exact numbers not reported)	Upper limb	0–2
Park JH, 2019 [33]	South Korea	Cross-sectional	34	Stroke (*n* = 31), TBI (*n* = 2), Brain tumour (*n* = 1)	Upper limb	0–3
Alcan V, 2017 [31]	Turkey	Cross-sectional	24	Stroke (n = 8), Healthy controls (*n* = 16)	Lower limb	1–3
Seth N, 2015 [30]	Canada	Cross-sectional	85	TBI (n = 18), Hemorrhagic stroke (n = 22), Healthy controls (*n* = 45)	Upper limb	0–3
Kim KS, 2011 [29]	South Korea	Cross-sectional	15	Stroke (*n* = 15)	Upper limb	1–2
Zupan B, 1998 [45]	Slovenia	Cross-sectional	98	SCI (*n* = 98)	Lower limb	0–4 #

Abbreviations: MAS, Modified Ashworth Scale; TBI, traumatic brain injury; CP, cerebral palsy; HIE, hypoxic-ischaemic encephalopathy; SCI, spinal cord injury; RCT, randomised controlled trial; *n*, number of participants. Note: * Sample sizes are reported as the total number of enrolled participants, with patients and healthy controls specified separately where applicable. # The study by Zupan et al. [45] used the original Ashworth Scale (AS), not the Modified Ashworth Scale (MAS).

**Table 2:** Summary of artificial intelligence (AI) methodology and performance.

Subgroup	First Author, Year	AI Methods	Input Data	AI Output	AI Performance	Comparator
**Discrimination between Spastic and Healthy Individuals**						
	Alcan V, 2017 [31]	FLC (ANFIS)	EMG-based (wavelet features)	Spasticity diagnosis (spastic/non-spastic/uncertain)	96% accuracy, 100% sensitivity, 94% specificity	MAS
	Seth N, 2015 [30]	CML (LDA, KNN, DTW)	Kinematic (3D force + position)	Healthy vs. patient with brain injury	DTW + KNN 85% accuracy	MAS
**Assessment of Spasticity Severity**						
	Zhou J, 2026 [44]	CML (NBC, KNN, DTC, RFC, ETC, GBDT, CBC, ABC, SVM), DL (BPNN)	EIM spectra (IMCM features)	Healthy vs. stroke; correlation with MAS	BPNN 88.6% accuracy; IMCM-S R^2^ = 0.87–0.90 with MAS	MAS
	Wu L, 2026 [43]	CML (KNN, RF, SVM, CatBoost)	EMG-based (7 features)	Spasticity severity (MAS 1–3)	CatBoost 87% accuracy	MAS
	Rezende A, 2025 [42]	CML (SVM, RF, KNN)	EMG-based (10 features)	Spasticity severity (MAS 0–3)	SVM 93% accuracy	MAS
	Li W, 2025 [41]	DL (CNN)	Triboelectric signals (TENG-SPA voltage, biomechanical)	Finger spasticity (MAS 0–4); arm function (FMA)	CNN 93% accuracy; MAS R^2^ = 0.85; FMA R^2^ = 0.88	MAS, FMA
	Lee IJ, 2024 [24]	CML (SVM, LDA, MLP, KNN, RF)	Biomechanical (19 IMUs + pressure ball)	Spasticity severity (3 levels) across 4 joints	SVM F1: elbow 0.95, wrist 0.92, thumb 0.95, fingers 0.90	MAS
	Wang C, 2024 [40]	CML (MLR, SVR, RF), DL (LSTM, FFNN),	Multimodal (sEMG + angle + torque)	Continuous spasticity severity score (0–4)	LSTM 89% accuracy; integrated index R = 0.86 with MAS	MAS, TSRT
	Daud MIA, 2024 [39]	CML (LDA, DT, SVM, KNN)	Kinetic (MMG signals; 25 features)	Spasticity severity (MAS 0–3)	KNN 91% accuracy	MAS
	Yee J, 2023 [25]	CML (GNB, DT, RF, XGBoost, SVM, Logical-SVM-RF)	Multimodal (kinematic + kinetic + sEMG)	Spasticity severity (MAS 0–4)	Logical-SVM-RF 91% accuracy	MAS
	Ye F, 2021 [22]	CML (BPNN)	EMG-based (5 features)	Arm function (FMA); spasticity severity (MAS)	BPNN correlations: FMA r > 0.9, MAS r = 0.80–0.92	MAS, FMA
	Wang C, 2021 [38]	CML (GA, EEMD, SVR)	Multimodal (sEMG + angle + torque)	Spasticity Assessment Score (0–4)	SAS R = 0.86 with MAS; R = −0.89 with TSRT	MAS, TSRT
	Chen Y, 2021 [37]	CML (KNN, SVM, RF, MLP)	Multimodal (sEMG + IMU)	Spasticity severity (MAS 0–2)	RF F1 = 0.95	MAS
	Yu S, 2020 [36]	FLC (ANFIS)	EMG-based (20 features reduced to 9 PCs)	Continuous spasticity score (0–2)	ANFIS R^2^ = 0.96, RMSE = 0.13	MAS, TSRT
	Kim JY, 2020 [35]	CML (DT, RF, SVM, LDA, MLP)	Kinematic (IMU accelerometer + gyroscope)	Elbow spasticity severity (MAS 0–4)	RF up to 95% accuracy	MAS
	Zhang X, 2019 [34]	CML (ULR,MLR,SVR)	Multimodal (sEMG + IMU)	Continuous spasticity score (0–3)	SVR fusion MSE = 0.059, R^2^ = 0.93	MAS
	Puzi AA, 2019 [32]	CML (Linear SVM, LDA, Weighted KNN)	Kinetic (torque-angle signals; 4 features)	Spasticity severity (MAS 0,1,2)	Linear SVM 84% accuracy	MAS
	Park JH, 2019 [33]	DL (MLP)	Kinetic (9 biomechanical parameters)	Spasticity severity (MAS 0–3)	MLP 82% agreement (kappa = 0.74)	MAS
	Kim KS, 2011 [29]	CML (K-means)	EMG-based (sEMG + angle; TSRT)	Spasticity severity (mild, moderate, severe based on TSRT)	TSRT clustering r = −0.95 with severity	MAS, TSRT
	Zupan B, 1998 [45]	CML (C4.5 decision tree, k-NN, LDA)	EMG-based (sEMG features; 26 features)	Spasticity severity (AS < 2 vs. AS ≥ 2, binary classification)	C4.5 up to 74.0% accuracy; k-NN up to 75.6% accuracy	AS
**Prediction of Future Risk of Spasticity**						
	Chen L, 2024 [46]	CML (MARS, AdaBoost, SVM, RF, GLM)	Clinical-only	To determine if spasticity develops at 6 months	RF AUC = 0.98; SVM AUC = 0.98	MAS
**Prediction of Response to Spasticity Treatment**						
	Chen H, 2026 [47]	CML (RF, SVM, XGBoost), DL (FFNN)	Multimodal (Clinical-only + Ultrasound biomarkers)	Response of patients to treatment (BoNT-A + vibration)	NN AUC = 0.87	MAS, FMA, BI

Abbreviations: ABC, AdaBoost classifier; ANFIS, adaptive neuro-fuzzy inference system; AS, Ashworth Scale; AUC, area under the curve; BI, Barthel Index; BoNT-A, botulinum toxin type A; BPNN, backpropagation neural network; CBC, CatBoost classifier; CML, conventional machine learning; CNN, convolutional neural network; DL, deep learning; DT, decision tree; DTC, decision tree classifier; DTW, dynamic time warping; EEMD, ensemble empirical mode decomposition; EIM, electrical impedance myography; EMG, electromyography; ETC, extremely randomized trees classifier; F1, F1-score; FFNN, feedforward neural network; FLC, fuzzy logic classifier; FMA, Fugl–Meyer Assessment; GA, genetic algorithm; GBDT, gradient boosting decision tree; GLM, generalised linear model; GNB, Gaussian naive Bayes; IMCM, impedance myography circle mapping; IMU, inertial measurement unit; KNN, k-nearest neighbours; LDA, linear discriminant analysis; LSTM, long short-term memory; MARS, multivariate adaptive regression spline; MAS, Modified Ashworth Scale; MLP, multilayer perceptron; MLR, multivariate linear regression; MMG, mechanomyography; NBC, naïve Bayes classifier; PC, principal component; R, correlation coefficient; R^2^, coefficient of determination; RF, random forest; RFC, random forest classifier; RMSE, root mean square error; SAS, Spasticity Assessment Score; sEMG, surface electromyography; SVM, support vector machine; SVR, support vector regression; TENG-SPA, triboelectric soft pneumatic actuator; TSRT, tonic stretch reflex threshold; ULR, univariate linear regression; XGBoost, extreme gradient boosting. Note: MAS ranges in the AI Output column refer to the classification task and may include healthy controls classified as MAS 0, where applicable. MAS ranges for the patient cohort alone are reported in Table 1.

**Table 3:** Summary of artificial intelligence (AI) validation, clinical implications, interpretability and limitations.

Subgroup	First Author, Year	Validation Method	External Validation	Clinical Implications	AI Interpretability	Key Limitations
**Discrimination between Spastic and Healthy Individuals**						
	Alcan V, 2017 [31]	Internal validation (No CV applied)	No	Fuzzy logic classifier discriminates spastic from healthy individuals using wavelet-transformed EMG	Intrinsic (fuzzy rule-based system)	Small sample (*n* = 24)
	Seth N, 2015 [30]	LOOCV	No	Dynamic time warping with KNN distinguishes MAS 0 from healthy, detecting subclinical abnormalities	Intrinsic (instance-based model: KNN; limited transparency)	Small sample for higher MAS; complex robotic setup
**Assessment of Spasticity Severity**						
	Zhou J, 2026 [44]	Nested 5-fold CV	No	IMCM-derived features provide a quantitative biomarker that correlates strongly with MAS	None	All-male sample (*n* = 83)
	Wu L, 2026 [43]	2:1 split + 3-fold CV	No	CatBoost classifier assesses spasticity severity using EMG features; multi-velocity training improves accuracy	None	Small sample (*n* = 11); exclusion of MAS 0 and 4
	Rezende A, 2025 [42]	70/30 split + 10-fold CV	No	Support vector machine assesses severity using both passive and active movements, capturing reflexive and voluntary components	None	Small sample (*n* = 15); uneven MAS distribution
	Li W, 2025 [41]	LOOCV + 90/10 split	No	Convolutional neural network assesses spasticity severity and motor function simultaneously using a single sensor	None	Small sample (*n* = 25)
	Lee IJ, 2024 [24]	LOSOCV	No	Support vector machine with RFE assesses spasticity severity in four upper limb joints during voluntary movement	Post hoc (model-specific feature importance)	Exclusion of MAS 3–4; complex 19-IMU setup
	Wang C, 2024 [40]	Train/test split	No	Integrated severity index provides continuous score correlating with MAS; dual-task reveals more severe spasticity	None	Small sample (*n* = 30); exclusion of MAS 4
	Daud MIA, 2024 [39]	90/10, 80/20, 70/30 splits + k-fold CV	No	K-nearest neighbours classifier assesses spasticity severity using MMG signals; feature selection improves accuracy and reduces training time	Intrinsic (instance-based model: KNN; limited transparency)	Uneven MAS distribution
	Yee J, 2023 [25]	90/10 split + 10-fold CV	No	Logical-SVM-RF hybrid model assesses all six MAS levels by combining logical rules with machine learning	Post hoc (SHAP)	Uneven MAS distribution
	Ye F, 2021 [22]	80/20 split + 5-fold CV	No	Backpropagation neural network assesses motor function and spasticity severity from EMG signals	None	Small sample (*n* = 29); poor cross-phase generalisation
	Wang C, 2021 [38]	Train/test split + 5-fold CV	No	Spasticity Assessment Score quantifies severity as continuous score with finer discrimination than MAS	None	Small sample (*n* = 26); exclusion of MAS 4
	Chen Y, 2021 [37]	80/20 split + 5-fold CV	No	Random forest with data fusion assesses spasticity severity during voluntary movement by combining EMG and motion signals	None	Small sample (*n* = 13); exclusion of MAS 3–4
	Yu S, 2020 [36]	LOOCV	No	Adaptive neuro-fuzzy inference system quantifies spasticity severity continuously with fewer passive stretches (4–5) compared to TSRT (20–56)	Intrinsic (neuro-fuzzy model: ANFIS)	Small sample (*n* = 15); age mismatch; exclusion of MAS 3–4
	Kim JY, 2020 [35]	LOOCV	No	Random forest classifier assesses spasticity severity using only IMU data, enabling low-cost wearable assessment	None	Uneven MAS distribution (MAS 4 *n* = 1)
	Zhang X, 2019 [34]	LOOCV	No	Support vector regression fusion quantifies spasticity severity by combining neurogenic and biomechanical biomarkers	Intrinsic (linear regression; coefficient-based interpretation)	Small sample (*n* = 24); exclusion of MAS 4; time-consuming protocol
	Puzi AA, 2019 [32]	5-fold CV	No	Linear support vector machine assesses spasticity severity using torque-angle features; catch position and post-catch stiffness are key discriminators	Intrinsic (linear SVM; coefficient-based interpretation)	Small sample (*n* = 25); exclusion of MAS 1+,3,4
	Park JH, 2019 [33]	Internal validation (No CV applied)	No	Multilayer perceptron learns clinical assessment patterns; catch angle, max resistance, speed are key biomechanical features	Post hoc (Layer-wise Relevance Propagation)	No train/test split or cross-validation; all data used for training; small MAS 3 sample (*n* = 3)
	Kim KS, 2011 [29]	Internal validation (No CV applied)	No	K-means clustering of TSRT quantifies spasticity severity with clearer separation between grades than MAS	Intrinsic (clustering: K-means; limited clinical interpretability)	No train/test split or cross-validation; small sample (*n* = 15); exclusion of MAS 0,3,4
	Zupan B, 1998 [45]	Stratified 10-fold CV	No	C4.5 and k-NN classifiers predict AS < 2 vs. AS ≥ 2 from sEMG features; thigh muscle activity during hip/knee manoeuvre was the strongest predictor	Intrinsic (C4.5 decision tree: rule-based, transparent; k-NN: instance-based, limited transparency; LDA: coefficient-based)	No external validation; small sample (*n* = 98); binary outcome (AS < 2 vs. AS ≥ 2); AS is not MAS; 1998 methods may not reflect contemporary AI/ML practice
**Prediction of Future Risk of Spasticity**						
	Chen L, 2024 [46]	70/30 split + 10-fold CV	No	Random forest and support vector machine predict 6-month spasticity risk using routine clinical data; FMA-UE strongest predictor	Post hoc (model-specific feature importance)	Exclusion of severe cognitive deficits
**Prediction of Response to Spasticity Treatment**						
	Chen H, 2026 [47]	70/30 split + 5-fold CV	No	Neural network predicts individual treatment response using ultrasound biomarkers; muscle thickness and echo intensity guide treatment decisions	None	Small sample for deep learning; 12-week follow-up may not capture long-term effects

Abbreviations: ANFIS, adaptive neuro-fuzzy inference system; AS, Ashworth Scale; C4.5, decision-tree algorithm; CV, cross-validation; EMG, electromyography; FMA, Fugl–Meyer Assessment; FMA-UE, Fugl–Meyer Assessment—Upper Extremity; IMCM, impedance myography circle mapping; IMU, inertial measurement unit; KNN, k-nearest neighbours; LDA, linear discriminant analysis; LOOCV, leave-one-out cross-validation; LOSOCV, leave-one-subject-out cross-validation; MAS, Modified Ashworth Scale; MMG, mechanomyography; RF, random forest; RFE, recursive feature elimination; sEMG, surface electromyography; SHAP, Shapley additive explanations; SVM, support vector machine; TSRT, tonic stretch reflex threshold.

**Table 4:** NHLBI study quality assessment.

Study	Q1	Q2	Q3	Q4	Q5	Q6	Q7	Q8	Q9	Q10	Q11	Q12	Q13	Q14	Quality Rating
Randomised Controlled Trial (NHLBI RCT Tool)															
Chen H, 2026 [47]	Y	Y	Y	Y	Y	Y	Y	Y	N	Y	Y	Y	Y	Y	Good
Observational Cohort and Cross-Sectional Studies (NHLBI Obs-Cohort/CS Tool)															
Wu L, 2026 [43]	Y	Y	NR	Y	N	N	NA	Y	Y	N	Y	NR	NA	N	Fair
Zhou J, 2026 [44]	Y	Y	NR	Y	N	N	NA	Y	Y	N	Y	NR	NA	N	Fair
Rezende A, 2025 [42]	Y	Y	NR	Y	N	N	NA	Y	Y	N	Y	NR	NA	N	Fair
Li W, 2025 [41]	Y	Y	NR	Y	N	N	NA	Y	Y	N	Y	NR	NA	N	Fair
Lee IJ, 2024 [24]	Y	Y	NR	Y	N	N	NA	Y	Y	N	Y	NR	NA	Y	Fair
Wang C, 2024 [40]	Y	Y	NR	Y	N	N	NA	Y	Y	N	Y	NR	NA	N	Fair
Daud MIA, 2024 [39]	Y	Y	NR	Y	N	N	NA	Y	Y	N	Y	NR	NA	N	Fair
Chen L, 2024 [46]	Y	Y	NR	Y	N	Y	Y	Y	Y	N	Y	NR	Y	Y	Fair
Yee J, 2023 [25]	Y	Y	NR	Y	N	N	NA	Y	Y	N	Y	NR	NA	N	Fair
Ye F, 2021 [22]	Y	Y	NR	Y	N	N	Y	Y	Y	Y	Y	NR	Y	N	Fair
Wang C, 2021 [38]	Y	Y	NR	Y	N	N	NA	Y	Y	N	Y	NR	NA	N	Fair
Chen Y, 2021 [37]	Y	Y	NR	Y	N	N	NA	Y	Y	N	Y	NR	NA	N	Fair
Yu S, 2020 [36]	Y	Y	NR	Y	N	N	NA	Y	Y	N	Y	NR	NA	N	Fair
Kim JY, 2020 [35]	Y	Y	NR	Y	N	N	NA	Y	Y	N	Y	NR	NA	N	Fair
Zhang X, 2019 [34]	Y	Y	NR	Y	N	N	NA	Y	Y	N	Y	NR	NA	Y	Fair
Puzi AA, 2019 [32]	Y	Y	NR	Y	N	N	NA	Y	Y	N	Y	NR	NA	N	Fair
Park JH, 2019 [33]	Y	Y	NR	Y	N	N	NA	Y	Y	N	Y	NR	NA	N	Fair
Alcan V, 2017 [31]	Y	Y	NR	Y	N	N	NA	Y	Y	N	Y	NR	NA	N	Fair
Seth N, 2015 [30]	Y	Y	NR	Y	N	N	NA	Y	Y	N	Y	NR	NA	N	Fair
Kim KS, 2011 [29]	Y	Y	NR	Y	N	N	NA	Y	Y	N	Y	NR	NA	N	Fair
Zupan B, 1998 [45]	Y	Y	NR	Y	N	N	NA	Y	Y	N	Y	NR	NA	N	Fair

Abbreviations: Y = Yes; N = No; NR = Not Reported; NA = Not Applicable; RCT = Randomised Controlled Trial; Obs-Cohort/CS = Observational Cohort and Cross-Sectional Studies.

**Table 5:** Summary of future research priorities.

Priority Area	Key Focus
External validation and generalisability	Multicentre datasets, diverse populations, improved representativeness
Study design robustness	Larger prospective cohorts, appropriate sample size, longitudinal prediction, reduced overfitting
Clinical translation	Workflow integration, EHR compatibility, user-friendly tools, real-time ML-simulation systems
Interpretability and trust	Transparent models, explainability methods for black-box AI, regulatory alignment and clinical trust

## Data Availability

No new data were created or analysed in this study. Data sharing is not applicable.

## Abbreviations

The following abbreviations are used in this manuscript:

AI	artificial intelligence
ANFIS	adaptive neuro-fuzzy inference systems
AUC	area under the curve
CML	conventional machine learning
CP	cerebral palsy
DL	deep learning
EIM	Electrical Impedance Myography
FFNNs	feedforward neural networks
FLCs	fuzzy logic classifiers
FMA	Fugl–Meyer Assessment
IMCM	Impedance Myography Circle Mapping
IMUs	inertial measurement units
KNN	k-nearest neighbours
LSTM-SVR	Long Short-Term Memory-Support Vector Regression
LSTMs	long short-term memory networks
MAS	Modified Ashworth Scale
mBI	modified Barthel Index
MLPs	multilayer perceptrons
MMG	mechanomyography
mRS	modified Rankin Scale
MS	multiple sclerosis
NHLBI	National Heart, Lung, and Blood Institute
NIHSS	National Institutes of Health Stroke Scale
PCC	Population, Concept, Context
PRISMA-ScR	Preferred Reporting Items for Systematic Reviews and Meta-Analyses extension for Scoping Reviews
PROBAST + AI	Prediction model Risk Of Bias Assessment Tool for Artificial Intelligence
RCTs	randomised controlled trials
RF	random forests
SCI	spinal cord injury
sEMG	surface electromyography
SVM	support vector machines
TBI	traumatic brain injury
XGBoost	Extreme Gradient Boosting

## Appendix A

**Table A1:** Search strategies for PubMed, Embase, CINAHL, and Web of Science.

Database	Step	Search Terms
PubMed	#1	“Muscle Spasticity”[Mesh] OR spastic[tiab] OR hyperton[tiab]
	#2	“Artificial Intelligence”[Mesh] OR “Machine Learning”[Mesh] OR “Deep Learning”[Mesh] OR “Neural Networks, Computer”[Mesh] OR “Natural Language Processing”[Mesh] OR “Support Vector Machine”[Mesh] OR AI[tiab] OR “machine learning”[tiab] OR “deep learning”[tiab] OR “neural network”[tiab] OR “support vector machine”[tiab] OR “random forest”[tiab] OR “convolutional neural network”[tiab] OR “reinforcement learning”[tiab] OR “computer vision”[tiab] OR “predictive model*”[tiab] OR “decision support”[tiab]
	#3	#1 AND #2
Embase	#1	‘muscle spasticity’/exp OR spastic:ti,ab OR hyperton:ti,ab
	#2	‘artificial intelligence’/exp OR ‘machine learning’/exp OR ‘deep learning’/exp OR ‘neural network’/exp OR ‘natural language processing’/exp OR ‘support vector machine’/exp OR ‘ai’:ti,ab OR ‘machine learning’:ti,ab OR ‘deep learning’:ti,ab OR ‘neural network’:ti,ab OR ‘support vector machine’:ti,ab OR ‘random forest’:ti,ab OR ‘convolutional neural network’:ti,ab OR ‘reinforcement learning’:ti,ab OR ‘computer vision’:ti,ab OR ‘predictive model*’:ti,ab OR ‘decision support’:ti,ab
	#3	#1 AND #2
CINAHL	#1	TI (“Muscle Spasticity” OR spastic* OR hyperton*) OR AB (“Muscle Spasticity” OR spastic* OR hyperton*)
	#2	TI (“Artificial Intelligence” OR “Machine Learning” OR “Deep Learning” OR “Neural Networks” OR “Natural Language Processing” OR “Support Vector Machine” OR AI OR “machine learning” OR “deep learning” OR “neural network” OR “support vector machine” OR “random forest” OR “convolutional neural network” OR “reinforcement learning” OR “computer vision” OR “predictive model*” OR “decision support”) OR AB (“Artificial Intelligence” OR “Machine Learning” OR “Deep Learning” OR “Neural Networks” OR “Natural Language Processing” OR “Support Vector Machine” OR AI OR “machine learning” OR “deep learning” OR “neural network” OR “support vector machine” OR “random forest” OR “convolutional neural network” OR “reinforcement learning” OR “computer vision” OR “predictive model*” OR “decision support”)
	#3	#1 AND #2
Web of Science	#1	TS = (“Muscle Spasticity” OR spastic* OR hyperton*)
	#2	TS = (“Artificial Intelligence” OR “Machine Learning” OR “Deep Learning” OR “Neural Networks” OR “Natural Language Processing” OR “Support Vector Machine” OR AI OR “machine learning” OR “deep learning” OR “neural network” OR “support vector machine” OR “random forest” OR “convolutional neural network” OR “reinforcement learning” OR “computer vision” OR “predictive model*” OR “decision support”)
	#3	#1 AND #2

Note: # indicates the search-set number, used to combine search lines (e.g., #1 AND #2). * Indicates truncation, allowing retrieval of all terms beginning with the preceding letters (e.g., spastic* retrieves spastic, spasticity).

**Table A2:** Eligibility criteria (PCC framework).

Domain	Inclusion Criteria	Exclusion Criteria
Population	Adults (≥18 years) with spasticity due to upper motor neuron lesions (e.g., stroke, cerebral palsy, spinal cord injury, traumatic brain injury, multiple sclerosis, neurodegenerative or acquired CNS disorders) Spasticity assessed, measured, predicted, or managed as an outcome	Non-spastic hypertonia only (e.g., rigidity, dystonia, non-neural contracture) Spasticity not formally assessed Paediatric or mixed populations without separable adult data
Concept	Studies applying AI/ML as a core method for spasticity evaluation, prediction, or management (e.g., ML, deep learning, neural networks, SVM, random forest, CNN, NLP, fuzzy logic, computer vision, decision support)	No AI/ML methods Conventional statistics only (e.g., regression, t-tests, ANOVA) Rule-based/deterministic models without learning No spasticity-specific outcomes
Context	Clinical, rehabilitation, outpatient, community, home-based, or tele-rehabilitation settings Human studies with ≥5 participants (development, validation, pilot, feasibility)	Synthetic/simulated data only <5 human participants
Study Design	RCTs, non-randomised clinical studies, cohort, case–control, cross-sectional, observational, pilot/feasibility, or validation studies Peer-reviewed journal articles	Animal or cadaver studies Case reports (*n* < 5) Conference abstracts, reviews, editorials, commentaries, letters, protocols without results
Language	English	Non-English

**Table A3:** PROBAST + AI risk of bias assessment.

Study	Participants	Predictors	Outcomes	Analysis (Key Flaws)	Overall
Wu L, 2026 [43]	Low	Low	Low	High—Extremely small sample; info leakage; non-independent test; no calibration; no fairness	High
Zhou J, 2026 [44]	Low	Low	Low	High—Info leakage; male-only bias; no calibration; no fairness	High
Chen H, 2026 [47]	Low	Low	Low	High—No external validation; no calibration; AUC only; no fairness	High
Rezende A, 2025 [42]	Low	Low	Low	High—Extreme small sample; imbalance; info leakage; no calibration; no fairness	High
Li W, 2025 [41]	Low	Low	Low	High—Small sample; time-series leakage; CNN overfit; no calibration; no fairness	High
Chen L, 2024 [46]	Low	Low	Low	High—No calibration; no external validation; small test set; no fairness	High
Lee IJ, 2024 [24]	Low	Low	Low	High—Small sample; info leakage; high-dim mismatch; no calibration; no fairness	High
Wang C, 2024 [40]	Low	Low	Low	High—Small sample; info leakage; overfitting; no calibration; no fairness	High
Daud MIA, 2024 [39]	Low	Low	Low	High—Small sample; info leakage; small test set; no calibration; no fairness	High
Yee J, 2023 [25]	Low	Low	Low	High—Limited severe cases; no CV; info leakage; no calibration; no fairness	High
Ye F, 2021 [22]	Low	Low	Low	High—Sample size *n* = 29; info leakage; overlapping windows; no calibration; no fairness	High
Wang C, 2021 [38]	Low	Low	Low	High—Sample size *n* = 16; info leakage; repeated trials violate independence; no calibration; no fairness	High
Chen Y, 2021 [37]	Low	Low	Low	High—Sample size *n* = 13; info leakage; overlapping windows; no calibration; no fairness	High
Yu S, 2020 [36]	Low	Low	Low	High—Sample size *n* = 15; info leakage; sliding window independence violation; no calibration; no fairness	High
Kim JY, 2020 [35]	Low	Low	Low	High—Small sample; imbalance; CV optimistic; no calibration; no fairness	High
Zhang X, 2019 [34]	Low	Low	Low	High—Sample size *n* = 24; info leakage; no calibration; no fairness	High
Puzi AA, 2019 [32]	Low	Low	Low	High—Small sample; no external validation; CV optimistic; no calibration; no fairness	High
Park JH, 2019 [33]	Low	Low	Low	High—Small sample; LOOCV optimistic; no calibration; no fairness	High
Alcan V, 2017 [31]	Low	Low	Low	High—Sample size *n* = 24; no CV; info leakage; binary only; no calibration; no fairness	High
Seth N, 2015 [30]	Low	Low	Low	High—Info leakage (feature selection pre-CV); no calibration; MAS regression poor; binary focus; no fairness	High
Kim KS, 2011 [29]	Low	Low	Low	High—Sample size *n* = 15; no external validation; no calibration; correlation alone insufficient; no fairness	High
Zupan B, 1998 [45]	Low	Low	Low	High—Small sample (*n* = 98); info leakage (feature selection before CV); no external validation; no calibration; AS not MAS; binary outcome only; no fairness assessment	High

Abbreviations: PROBAST + AI, Prediction model Risk Of Bias Assessment Tool for Artificial Intelligence; Low, low risk of bias; High, high risk of bias (one or more critical flaws).
